# The triglyceride glucose index trajectory is associated with hypertension: a retrospective longitudinal cohort study

**DOI:** 10.1186/s12933-023-02087-w

**Published:** 2023-12-15

**Authors:** Fengling Xin, Shuyou He, Yu Zhou, Xueni Jia, Yulong Zhao, Hui Zhao

**Affiliations:** 1 School of Public Health, Dalian Medical University, No.9, West Section of Lushunkou Road, Lushunkou District, Dalian, Liaoning, 116000 China; 2 Dalian Neusoft Institute of Information, No.8, Software Park Road, Ganjingzi District, Dalian, Liaoning, 116000 China; 3 Health Management Center of the Second Affiliated Hospital of Dalian Medical University, No.467, Zhongshan Rode, Shahekou District, Dalian, Liaoning, 116000 China

**Keywords:** Triglyceride glucose index, Hypertension, Long-term trajectories, Retrospective cohort

## Abstract

**Background:**

Previous studies have found that the triglyceride glucose index (TyG index) trajectories are associated with cardiovascular diseases. However, the association between the patterns of TyG index trajectories and risk for hypertension has not been investigated. In a longitudinal general population, we aimed to identify distinct TyG index trajectories over 12 years and describe their association with incidence of hypertension.

**Method:**

Of the 15,056 adults retrospectively recruited from the Physical Examination Center of the Second Affiliated Hospital of Dalian Medical University in northeast of China from 2011 to 2022. TyG index was calculated as ln (fasting TG [mg/dL] × FPG [mg/dL]/2) and the TyG index trajectories were developed using group-based trajectory modelling. Cox regression analysis was accomplished to assess the association between TyG index and incidence of hypertension.

**Results:**

The median age of the population was 38 years, and 7352 (48.83%) of the participants were men. Three distinct TyG index trajectories were identified: “low increasing” (N = 7241), “moderate increasing” (N = 6448), and “high stable” (N = 1367). Using “low increasing” trajectory as a reference, “moderate increasing” and “high stable” trajectory were associated with increased risk of hypertension (HR = 2.45; 95% CI 2.25–2.67 and HR = 3.88; 95% CI 3.48–4.33). After adjusting for baseline sex, age, diabetes, smoking, systolic blood pressure, diastolic blood pressure, BMI, cholesterol, high density lipoprotein cholesterol, low density lipoprotein cholesterol, blood glucose, triglyceride, urea, uric acid, and glomerular filtration rate, the HR were slightly attenuate in “moderate increasing” and “high stable” trajectories to 1.38 (95% CI 1.23–1.54) and 1.69 (95% CI 1.40–2.02) respectively. Meanwhile, similar results were observed in multiple sensitivity analyses. The HR of the “moderate increasing” and “high stable” trajectory groups were 2.63 (95% CI 2.30–3.00) and 4.66 (95% CI 3.66–5.93) in female, and 1.66 (95% CI 1.48–1.86) and 2.33 (95% CI 2.04–2.66) in male.

**Conclusions:**

Elevated TyG index at baseline and long-term TyG index trajectories were associated with the risk of hypertension. Early identification of increasing TyG index could provide insights for preventing hypertension later in life.

**Supplementary Information:**

The online version contains supplementary material available at 10.1186/s12933-023-02087-w.

## Introduction

Hypertension is a momentous cause of cardiovascular disease, especially in developing countries [1–3]. Meanwhile, the prevalence of hypertension keeps increasing globally [4–6]. According to the World Health Organization, 1.4 billion individuals worldwide suffer from high blood pressure, and there are approximately 270 million people in China [7, 8]. Hence, recognizing the high-risk factors of hypertension presents a chance for prompt intervention and mitigating the multitude of detrimental consequences of hypertension, specifically in terms of diminishing cardiovascular morbidity [9].

Recent studies have demonstrated that the triglyceride-glucose (TyG) index is correlated with incident hypertension [10–15] and associated with various cardiovascular diseases, including peripheral artery disease, coronary artery disease, stroke, myocardial infarction, carotid atherosclerosis, and arterial stiffness [16–22]. TyG is a composite indicator and has been regarded as a surrogate marker of insulin resistance, which is calculated as In (fasting triglycerides [TG, mg/dL] × fasting blood glucose [FBG, mg/dL]/2) [18, 20]. Previous studies evaluating the association of the TyG index and increased blood pressure and its relation to new-onset hypertension in general adult population [11]. These studies analyzed the TyG index from single time-point data, and little is known about the long-term TyG index and subsequent risk of hypertension. Exploring the relationship between the trajectory of TyG index fluctuates over time and subsequent risk of hypertension could identify patients who are at risk for developing hypertension, thereby enabling effective strategies to public health in primary hypertension prevention.

Consequently, we employed large-scale retrospective data recruited from 2011 to 2022 to identify various trajectories of the TyG index by group-based trajectory modeling (GBTM), and to estimate the correlation between TyG index trajectories and the prevalence of hypertension in the general populace.

## Methods

### Study population

**Fig. 1:**
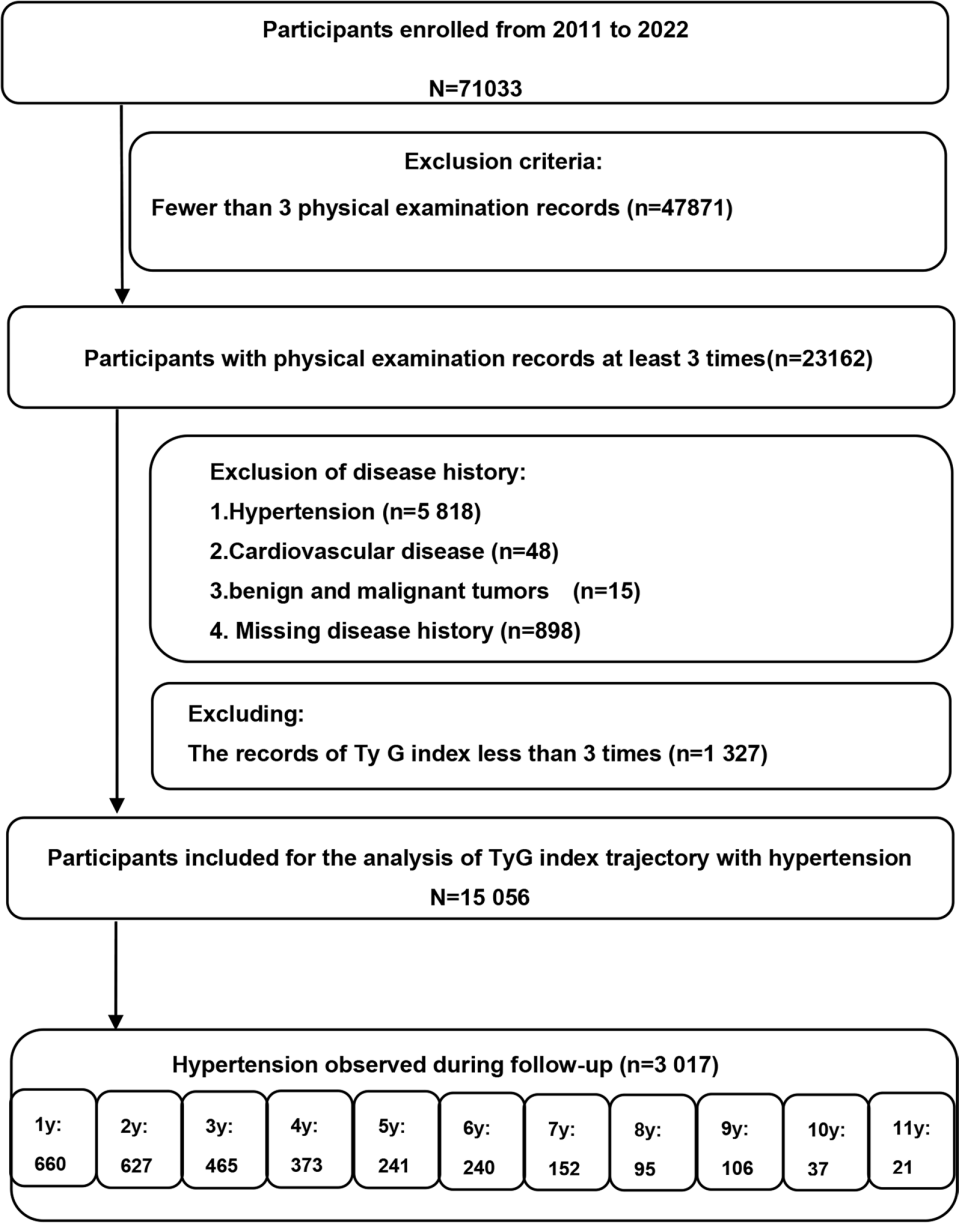
Flowchart of the study participants enrollment. y, year

The population was derived from the Physical Examination Center of the Second Affiliated Hospital of Dalian Medical University in Dalian, China. We retrospectively enrolled 71,033 participants, aged 19**–**92 years, from the health examination database from 2011 to 2022. All available data at every visit of all participants, including age, sex, height, weight, BMI, smoking, alcohol consumption status, medical history, and family history, were taken into account. For the purposes of this study, we excluded participants using the following criteria: Firstly, participants with fewer than 3 physical examination records (n = 47,871) were excluded. Secondly, we eliminated individuals who had been diagnosed with hypertension (n = 5818), a prior record of cardiovascular disease (n = 48), a previous history of both benign and malignant tumors (uterine fibroids, teratoma, lymphoma, thyroid cancer, meningioma, renal cysts, or acoustic neuroma) (n = 15), or missing the history of disease (n = 898) at baseline. Thirdly, we also excluded those with less than 3 TyG index measurements (n = 1327). Therefore, the remaining 15,056 participants with 59,999 measures were included for further analysis (Fig. 1). The earliest physical examination data of every participant was recorded as the baseline, and the following physical examination visits were considered as follow-up. The occurrence of hypertension during the follow-up was considered as the outcome event. The study was approved by the Ethics Committee of the Second Affiliated Hospital of Dalian Medical University, which waived the requirement for patient informed consent.

### Data collection and definition

Height and weight were measured using the automated instrument with participants standing straight and barefoot. BMI was calculated as weight (kg)/height (m)^2^. Family history included parental hypertension and diabetes. A smoker was defined as smoking continuously or cumulatively for more than 1 year and at least smoking 1 cigarette one day [23]_._ A drinker was defined as drinking for over 1 year and drinks on average ≥ 100 ml/d [23]. Systolic blood pressure (SBP) and diastolic blood pressure (DBP) were measured twice on the right arm using a sphygmomanometer after resting quietly for at least 5 min. Blood samples were collected after fasting for at least 8 h. Biochemical parameters, including blood glucose (FBG), triglyceride (TG), total cholesterol (TC), high-densitylipoprotein (HDL), low-densitylipoprotein (LDL), white blood cell (WBC), red blood cell (RBC), neutrophils (NE), platelet (PLT), haemoglobin (Hb), albumin, urea, creatinine (Cr) and uric acid (UA) were determined by standard laboratory techniques. The TyG index at each visit was calculated as ln (fasting TG [mg/dL] × FPG [mg/dL]/2) [18, 20]. Studies have shown that the calculation of eGFR was more consistent with Chinese individuals using the FAS formula [24]. Diabetes was defined as the fasting glucose level ≥ 126 mg/dL (≥ 7.0 mmol/L), HbA1c ≥ 6.5% or a self-reported physician diagnosis of diabetes or any use of antidiabetic medications [25]. Hypertension was defined as SBP ≥ 140 mmHg, DBP ≥ 90 mmHg, or taking antihypertensive drugs according to clinical data or self-report [26].

### Statistical analysis

Statistical analyses were performed using R (version 4.2.2), Stata (version 16), and GraphPad Prism 9 software. Continuous variables with normal distributions were described as mean ± standard deviation (SD), while skewed distributions were presented using medians with interquartile ranges. Normal distribution data was tested using Kolmogorov Smirnov test, and the Mann–Whitney U test or Kruskal–Wallis test was used for skewed distributions. Categorical variables were described as percentages and compared using chi-square test. A two-sided P value of < 0.05 was recognized as statistically significant.

Group-Based trajectory modeling (GBTM) was used to identify TyG trajectory subgroups that shared similar growth patterns of the TyG index over time [27, 28]. The most suitable trajectory, ranging from 2 to 5 groups, was determined based on Bayesian information criteria (BIC), Akaike information criterion (AIC), entropy, and average posterior probability. Specifically, the optimal number of trajectory groups was determined by: (1) the model with the minimum BIC absolute value; (2) accounting for at least 5% of the total membership in each trajectory; and (3) higher average posterior probabilities (> 0.70). Ultimately, we considered that three groups were the best fitting trajectory groups, and Additional file 1: Table S1 provides detailed information on the modeling strategies employed. All final models effectively classify participants into trajectory groups and demonstrate strong discriminatory ability: the average probabilities for each member are 0.93, 0.90, and 0.93, respectively.

Cox proportional hazards model was calculated to estimate the association between different TyG index trajectories and hypertension risk, presented as hazard ratio (HR) and 95% confidence intervals (95%CI). Two multivariate models were conducted to adjusted for potential confounding elements: Model 1 was adjusted for age and sex. Model 2 was further adjusted for baseline diabetes, smoking, SBP, DBP, BMI, TC, HDL, LDL, TG, FBG, urea, UA and eGFR. We further used the restricted cubic spline regression model, with 5 knots, to more thoroughly assess the dose–response relationship between the TyG index and new-onset hypertension. Additionally, the Kaplan–Meier method was used to estimate the incidence curves of hypertension over the total follow-up period.

To further confirm the robustness of our results, we conducted 4 sensitivity analyses. Firstly, to exclude the influence of medications, we excluded participants who used any antidiabetic or lipid-lowering medications to assess whether the results were affected. Secondly, to exclude the potential bias of missing data, we completed the missing data with multiple imputations. Thus, we created five imputed data sets, selecting the most appropriate dataset to input the missing variables. Additionally, all variables missing at baseline and blood pressure missing during follow-up were presented in Additional file 1: Fig. S1. Thirdly, to reduce the difference between all trajectories, we conducted multigroup propensity scores (Additional file 1: Fig. S2). Finally, we excluded participants who developed hypertension at second visit to minimize potential reverse causality, considering short length of follow-up may influence the outcome.

Furthermore, to gain a more comprehensive understanding of the potential relationship between TyG index trajectories and the risk of hypertension, we also conducted subgroup analyses based on different gender, age, and BMI levels.

## Results

### Study population

In total, 15,056 eligible individuals were included, the median age was 38 (30**–**47) years, and 7352 (48.83%) were male. The median TyG index was 8.44 at baseline. On average, all participants had a mean of 3.99 individual physical examination records and a mean 4.13 years of follow up. During follow-up, 3017 participants who were identified as having hypertension. The baseline characteristics were described according to the quartile range of the TyG index in Additional file 1: Table S2. Compared with the participants with lower baseline TyG quartiles, who with higher TyG were more likely to have higher blood pressure, BMI, FPG, TC, TG, LDL, and lower HDL.

### Latent TyG index trajectories

**Fig. 2:**
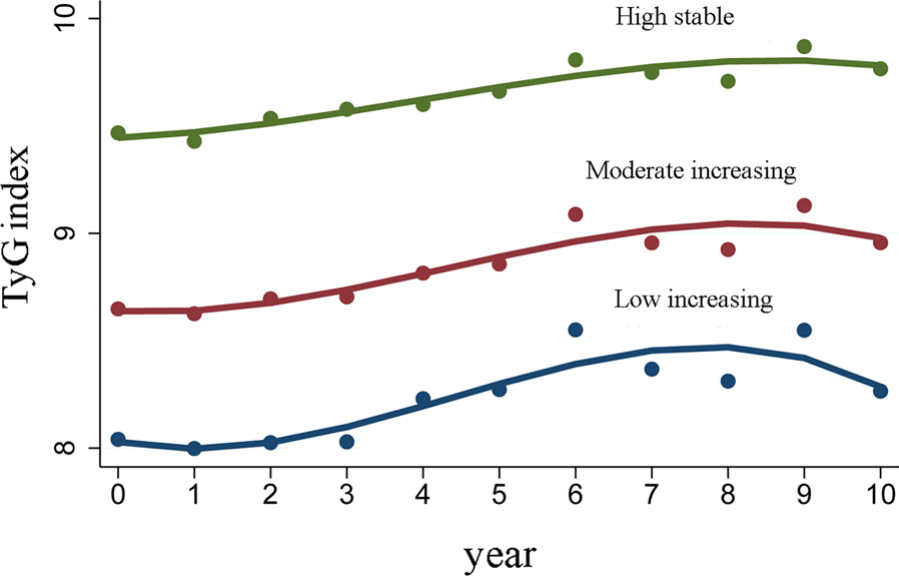
Long-term TyG index trajectories derived from Group-Based trajectory modeling

We identified three distinct TyG index trajectories among the 15,056 participants: “low increasing” (n = 7241, 48.09%), “moderate increasing” (n = 6448, 42.83%), and “high stable” (n = 1367, 9.08%) (Fig. 2). The baseline characteristics of each trajectory are shown in Table 1. Females were overrepresented in the “low increasing” trajectory (68.82%), while the “moderate increasing” and “high stable” trajectory contained more males. Moreover, there was an upward trend in the median age, from 34.00 (28.00**–**43.00) years in the “low increasing” trajectory to 45.00 (36.00**–**52.00) years of the “high stable” trajectory. The levels of TyG, BMI, FPG, TC, TG, LDL, diabetes, smoking, drinking, and family history of diabetes were higher in the “high stable” trajectory compared to other two trajectories, and HDL was lower in the “high stable” trajectory. However, the family of hypertension was similar in three trajectories. Notably, participants in the “high stable” trajectory had higher SBP and DBP levels than their counterparts in the “low increasing” and “moderate increasing” trajectory.

**Table 1:** Baseline characteristics of participants in different TyG index trajectories

Characteristics	Low increasing	Moderate increasing	High stable	*p*
n	7241	6448	1367	
Age, years^a^	34.00 (28.00–43.00)	42.00 (32.00–50.00)	45.00 (36.00–52.00)	< 0.01
Male (%)^b^	2,258 (31.18)	3,976 (61.67)	1,118 (81.78)	0.00
TyG index^a^	8.09 (7.85–8.32)	8.70 (8.47–8.96)	9.48 (9.21–9.80)	0.00
SBP (mmHg)^a^	116.00 (108.00–125.00)	122.00 (114.00–130.00)	125.00 (117.00–131.00)	< 0.01
DBP (mmHg)^a^	70.00 (64.00–76.00)	74.00 (68.00–80.00)	77.00 (70.00–82.00)	< 0.01
Height (cm)^a^	166.00 (161.00–172.00)	170.00 (164.00–176.00)	172.00 (167.00–177.00)	< 0.01
Weight (kg)^a^	60.00 (54.00–68.00)	71.00 (63.00–80.00)	78.00 (70.00–85.00)	0.00
BMI (kg/cm^2^)^a^	21.88 (20.09–23.89)	24.57 (22.58–26.70)	26.13 (24.22–28.09)	0.00
WBC (10^9/L)^a^	5.66 (4.86–6.60)	6.26 (5.38–7.32)	6.75 (5.83–7.95)	< 0.01
RBC (10^12/L)^a^	4.58 (4.34–4.91)	4.89 (4.57–5.20)	5.06 (4.80–5.31)	0.00
Hb (g/L)^a^	136.00 (128.00–147.00)	149.00 (137.00–158.00)	155.00 (146.00–162.00)	0.00
NE (10^9)^a^	3.17 (2.57–3.89)	3.51 (2.89–4.28)	3.78 (3.11–4.60)	< 0.01
PLT (10^9)^a^	225.00 (195.00–259.00)	225.00 (193.10–259.00)	220.00 (190.00–255.00)	0.01
Alb (g/L)^a^	46.20 (44.56–47.90)	46.50 (44.80–48.20)	47.00 (45.30–48.70)	< 0.01
UREA (mmol/L)^a^	4.47 (3.73–5.29)	4.79 (4.04–5.59)	4.94 (4.25–5.70)	< 0.01
Cr (µmol/L)^a^	59.00 (52.00–71.00)	69.00 (58.00–78.88)	71.59 (63.00–79.84)	< 0.01
UA (µmol/L)^a^	278.22 (236.00–336.00)	346.22 (287.00–410.00)	392.00 (334.00–453.99)	0.00
FPG (mmol/L)^a^	5.20 (4.97–5.47)	5.48 (5.20–5.79)	5.84 (5.44–6.67)	0.00
TC (mmol/L)^a^	4.44 (3.96–4.95)	4.90 (4.37–5.48)	5.19 (4.64–5.78)	0.00
TG (mmol/L)^a^	0.78 (0.63–0.97)	1.37 (1.08–1.75)	2.69 (2.05–3.54)	0.00
HDL (mmol/L)^a^	1.44 (1.25–1.65)	1.18 (1.03–1.37)	1.00 (0.89–1.14)	0.00
LDL (mmol/L)^a^	2.43 (2.04–2.86)	2.91 (2.48–3.41)	2.81 (2.29–3.33)	0.00
eGFR (ml/min)^a^	114.99 (103.75–127.69)	108.83 (97.55–120.24)	108.06 (97.47–120.24)	< 0.01
diabetes, n (%)^b^	14 (0.19)	95 (1.47)	143 (10.46)	< 0.01
smoking (%)^b^	127 (1.75)	338 (5.24)	132 (9.65)	< 0.01
Drinking (%)^b^	13 (0.18)	40 (0.62)	10 (0.73)	< 0.01
family history of hypertension (%)^b^	335 (4.63)	313 (4.85)	50 (3.66)	0.16
family history of diabetes (%)^b^	166 (2.29)	190 (2.95)	50 (3.66)	0.00

Non-normally distributed variables are expressed as the median (interquartile range) or as numbers (percentage)

BMI, body mass index; HDL, high density lipoprotein; LDL, low density lipoprotein; SBP, systolic blood pressure; DBP, diastolic blood pressure; WBC, white blood cell; RBC, red blood cell; NE, neutrophils; Hb, haemoglobin; Alb albumin; PLT, platelet; CHOL, cholesterol; Cr, creatinine; UA, uric acid; TC, total cholesterol; FPG, fasting plasma glucose; TG, triglyceride glucose; TyG, Triglyceride glucose index = ln(fasting TG [mg/dL] × fasting plasma glucose [mg/dL]/ 2); eGFR, Glomerular filtration rate;.

^a^Data are given as median (interquartile range)

^b^Data are expressed as number (percentage)

### Association between TyG index trajectory and risk of hypertension

The occurrence rate of hypertension in three trajectory groups presented an increasing trend, which were 10.91% (n = 790), 26.21% (n = 1,690), and 39.28% (n = 537) in the “low increasing”, “moderate increasing”, and “high stable” trajectory groups, respectively (Table 2). Additional file 1: Fig. S3 showed the different incidence rates of hypertension in every follow-up year for all the trajectories. Overall, the incidence rates of hypertension in the “high stable” and “moderate increasing” trajectories were higher than the “low increasing” trajectory during all follow up years, and the “high stable” trajectory had the highest ratio of hypertension incidence at each time point. Meanwhile, more higher hypertension risk was found in the earlier years than in the longer timeframe, and the occurrence of hypertension in each trajectory group showed an inverted J shape.

**Table 2:** Association between TyG index trajectory with incidence of hypertension

Variables	Low increasing	Moderate increasing	High stable	*p*
Event/total (%)	790/7241 (10.91)	1690/6,448 (26.21)	537/1367 (39.28)	
Unadjusted HR (95% CI)	Reference	2.45 (2.25–2.67)	3.88 (3.48–4.33)	< 0.01
Model 1	Reference	1.77 (1.62–1.93)	2.36 (2.10–2.65)	< 0.01
Model 2	Reference	1.38 (1.23–1.54)	1.69 (1.40–2.02)	< 0.01

Model 1 was adjusted for baseline age and sex

Model 2 further adjusted for model 1 covariates plus baseline diabetes, smoking, systolic blood pressure, diastolic blood pressure, BMI, high density lipoprotein cholesterol, low density lipoprotein cholesterol, blood glucose, triglyceride, cholesterol, urea, uric acid, glomerular filtration rate

Table 2 outlines the association between TyG index trajectory and hypertension risk. Taking the “low increasing” trajectory as the reference, the HR (95% CI) in the “moderate increasing” and “high stable” trajectories were 2.45 (2.25**–**2.67) and 3.88 (3.48**–**4.33), respectively. These associations were moderately attenuated when adjusted for age and sex, the HR and 95% CI of “moderate increasing” and “high stable” trajectories were 1.77 (1.62**–**1.93) and 2.36 (2.10**–**2.65). Further adjustment for potential confounders of the TyG index and hypertension showed that the HR (95% CI) of “moderate increasing” and “high stable” trajectories were 1.38 (1.23**–**1.54) and 1.69 (1.40**–**2.02), respectively. The positive association remained statistically significant.

**Fig. 3:**
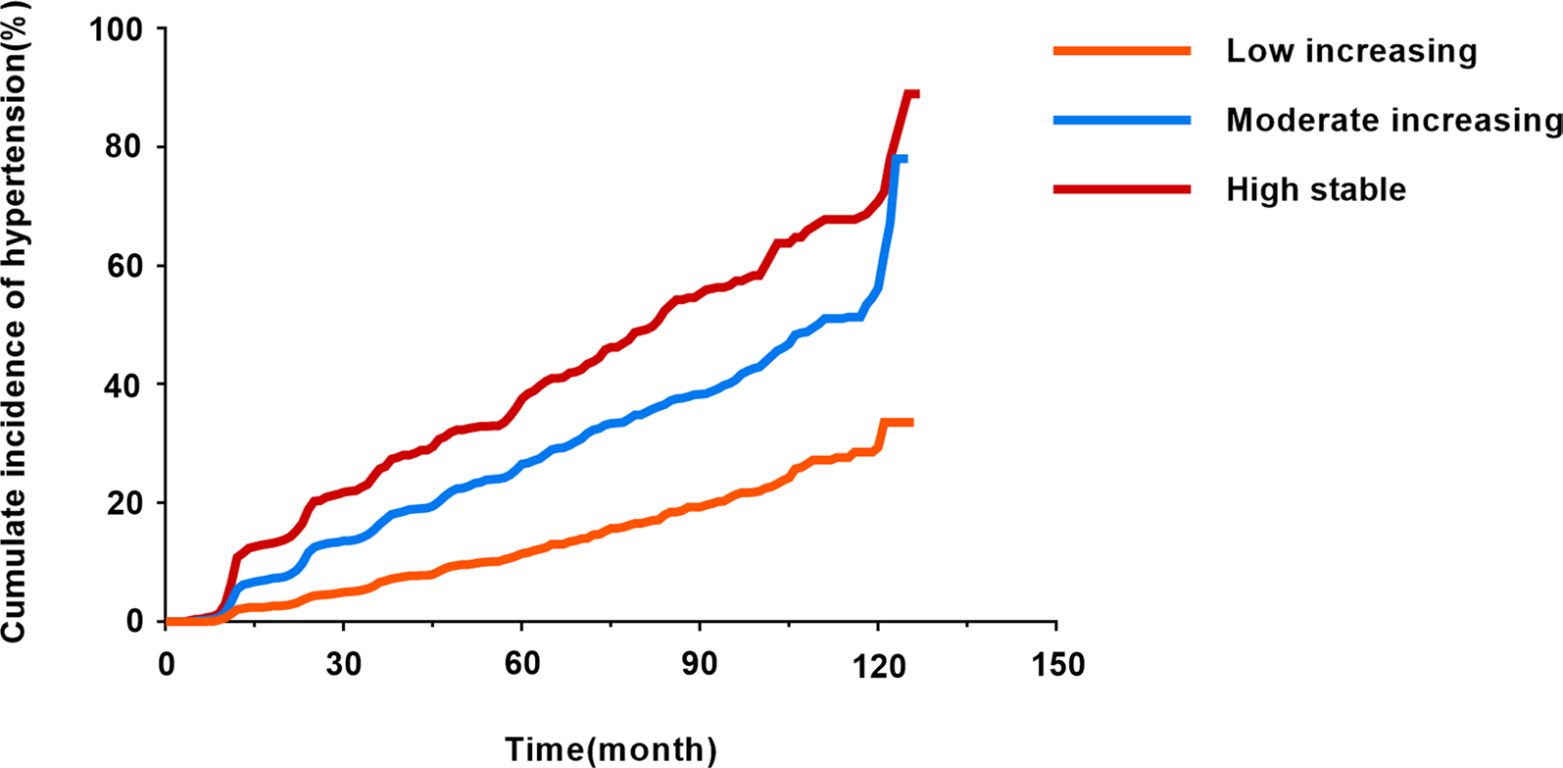
Kaplan–Meier estimation of cumulative incidence curves of hypertension in different TyG index trajectories

Furthermore, the Kaplan–Meier curve showed that the slope of the tangent line at the same time in the “moderate increasing” and “high stable” trajectory groups is greater compared to the “low increasing” trajectory group. This indicates that means the cumulative incidence of hypertension increased with the TyG index trajectory group (Fig. 3).

### Sensitivity analysis

Four sensitivity analyses were performed, and the results were consistent (Table 3). First, after excluding the participants who were taking any antidiabetic or lipid lowering medications, the TyG index trajectories were still significantly associated with hypertension incidence (Additional file 1: Fig. S4A). Similarly, when using the complete data and inputting the missing data with multiple imputation, it was proved that the association of hypertension incidence was also significant in the TyG index trajectories (Additional file 1: Fig. S4B). Moreover, using data disposed by multi-group propensity scores, similar results were obtained (Additional file 1: Fig. S4C). Additionally, excluding the participants who were diagnosed with hypertension at the second visit did not lead to substantial changes in the results (Additional file 1: Fig. S4D).

**Table 3:** Sensitivity analysis between the TyG trajectory and risk of hypertension

	Low increasing	Moderate increasing	High stable
Unadjusted			
Sensitivity^1^	1 (reference)	2.43 (2.23–2.64)	3.87 (3.46–4.33)
Sensitivity^2^	1 (reference)	2.44 (2.25–2.66)	3.85 (3.45–4.29)
Sensitivity^3^	1 (reference)	1.48 (1.30–1.69)	2.73 (1.92–3.89)
Sensitivity^4^	1 (reference)	2.31 (2.07–2.58)	3.21 (2.76–3.73)
Model 1			
Sensitivity^1^	1 (reference)	1.76 (1.61–1.92)	2.41 (2.14–2.71)
Sensitivity^2^	1 (reference)	1.77 (1.62–1.93)	2.35 (2.10–2.64)
Sensitivity^3^	1 (reference)	1.49 (1.31–1.70)	2.50 (1.76–3.57)
Sensitivity^4^	1 (reference)	1.71 (1.52–1.91)	2.00 (1.71–2.34)
Model 2			
Sensitivity^1^	1 (reference)	1.37 (1.22–1.53)	1.69 (1.40–2.03)
Sensitivity^2^	1 (reference)	1.36 (1.23–1.51)	1.67 (1.42–1.97)
Sensitivity^3^	1 (reference)	1.40 (1.20–1.62)	2.51 (1.69–3.72)
Sensitivity^4^	1 (reference)	1.41 (1.22–1.63)	1.58 (1.23–2.03)

Sensitivity^1^: Diabetes mellitus and hypoglycemic agents are excluded

Sensitivity^2^: multiple imputation

Sensitivity^3^: propensity score matching

Sensitivity^4^: participants who developed hypertension at visit 2 were excluded

Model 1 was adjusted for baseline age and sex

Model 2 further Adjusted for model 1 covariates plus baseline diabetes, smoking, systolic blood pressure, diastolic blood pressure, BMI, high density lipoprotein cholesterol, low density lipoprotein cholesterol, blood glucose, triglyceride, cholesterol, urea, uric acid, glomerular filtration rate

### Stratified analysis

**Fig. 4:**
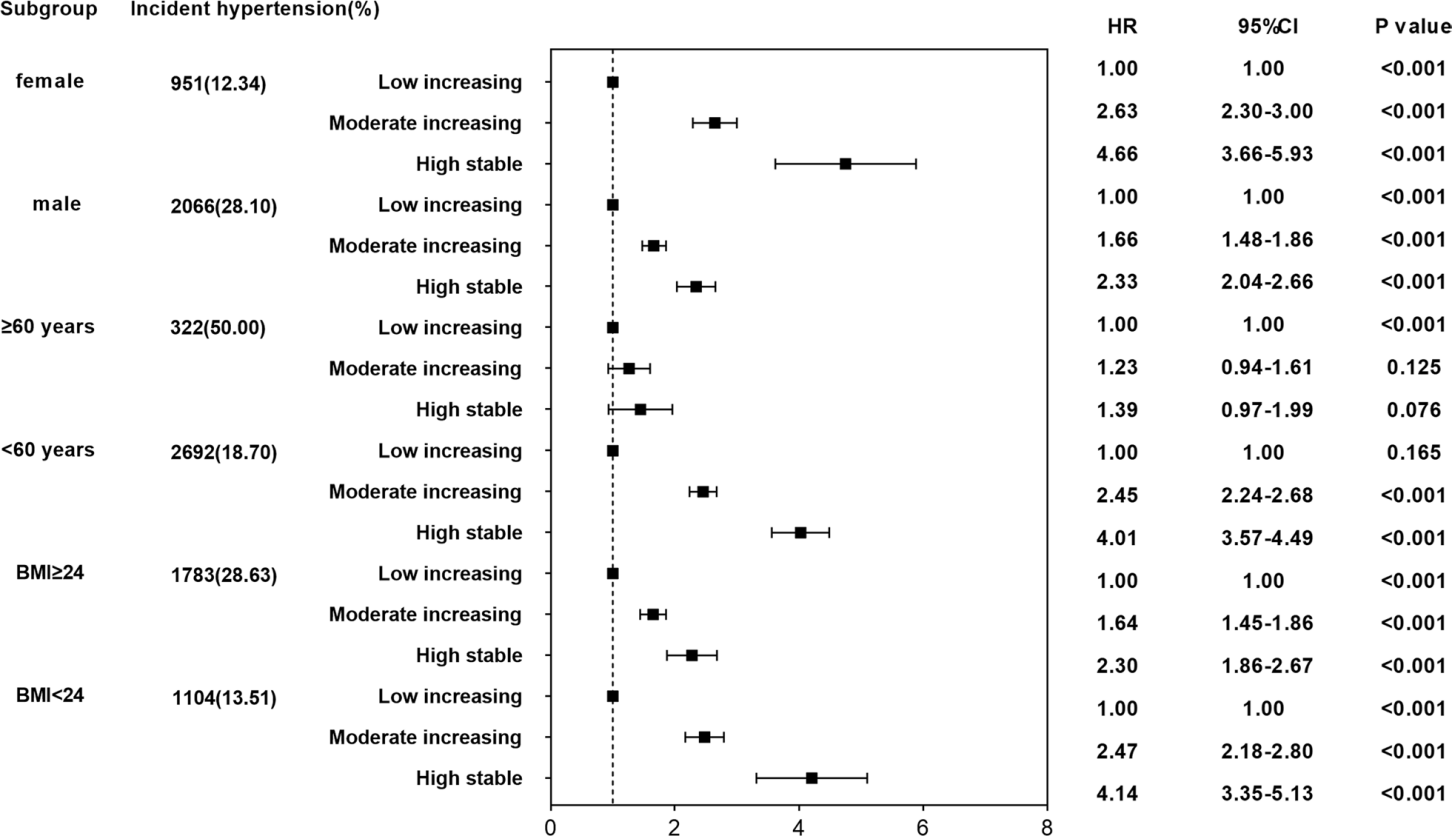
Adjusted HR for incident of hypertension in different trajectories by stratified analyses

Considering the potential effects of sex, age, and BMI, Fig. 4 shows the hypertension risk for participants stratified by sex (male or female), age (< 60 or ≥ 60 years), and BMI (< 24 or ≥ 24 kg/m^2^) status at baseline. For males, the incidence of hypertension was 28.10% throughout the follow-up period, while for females, it was lower at 12.34%. For males, the risk of outcome events in the “moderate increasing” and “high stable” trajectory groups were 1.66 (1.48**–**1.86) and 2.33 (2.04**–**2.66),respectively. On the other hand, for females, a higher risk was observed in the “moderate increasing” (HR = 2.63; 95% CI 2.30**–**3.00) and “high stable” trajectory groups (HR = 4.66; 95% CI 3.66**–**5.93).

In addition, participants younger than 60 (50.00%) showed significantly increased ratio of developing hypertension compared to those older than 60 (18.70%). Meanwhile, for participants younger than 60, a significant high risk was observed in the “moderate increasing” and “high stable” trajectories (HR = 2.45; 95%CI 2.24**–**2.68 and HR = 4.01; 95%CI 3.57**–**4.49). However, there was no statistically significant difference in the “moderate increasing” and “high stable” trajectories when compared with the “low increasing” trajectory, in the participants older than 60 years.

Notably, the prevalence of hypertension in participants with BMI ≥ 24 was twice higher than that in participants with BMI < 24 (28.63% *vs.* 13.51%). However, participants with BMI < 24 had increasing risk of hypertension in “moderate increasing” (HR = 2.47; 95% CI 2.18**–**2.80) and “high stable” (HR = 4.14; 95% CI 3.35**–**5.13) trajectories, respectively. While less pronounced, for participants with BMI ≥ 24, the HR and 95% CI of hypertension in “moderate increasing” and “high stable” trajectories were 1.64 (1.45**–**1.86) and 2.30 (1.86**–**2.67), respectively.

## Discussion

Our findings revealed the positive relationship between the TyG index and incidence of hypertension, which is consistent with previous studies [10–12]. We further identified three distinct trajectories of the TyG index using GBTM over a 12-year follow-up period, and a similar significant association was observed between the TyG index trajectories and the incidence of hypertension. Additionally, this relationship was consistent across different subgroups and in sensitivity analyses. To the best of our knowledge, this study is the first to demonstrate the relationship between TyG index trajectories and hypertension in a large, longitudinal population of general adults. These results confirmed that the TyG index is a crucial discriminator for the risk of hypertension. In clinical practice, individuals with stable and high-level of TyG index require to maintain healthy diet, regular exercise and early and intensive screening and management of risk factors to prevent hypertension and subsequent cardiovascular events.

Previous studies have confirmed the TyG index was associated with hypertension [10–13, 15, 29, 30]. Prior observational data from China Health and Retirement Longitudinal Study (CHARLS), which enrolled 8,209 participants, showed that compared to the lowest quartile of the TyG index, the highest quartile was associated with increased risks of stage 1 hypertension (OR 1.71, 95% CI 1.38**–**2.13), stage 2 (1.74, 1.27**–**2.38), isolated systolic hypertension (1.66, 1.31**–**2.11), isolated diastolic hypertension (2.52, 1.26**–**5.05), and systolic diastolic hypertension (1.65, 1.23–2.23), respectively[31]. Another large population-based study (n = 47,808) also reported TyG index significantly associated with hypertension in either total subjects or subjects separated into men and women [13]. These associations persist in cross-sectional studies in large general populations [30]. Our results are consistent with previous studies, the participants with higher TyG quartiles were more likely to have higher blood pressure, compared with the ones with lower baseline TyG quartiles (Table S2). We also assessed patients with hypertension at baseline who had higher TyG than the normotensive subjects (Table S3). Meanwhile, the restrictive cubic spline model showed that when the TyG index was greater than 8.44, the risk of incidence of hypertension was significantly and positively increased (p non-linearity < 0.001) (Additional file 1: Fig. S5). Many studies also confirmed that the TyG index is positively related to high blood pressure in specific participants. Huang et al. reported that a higher TyG index is associated with elevated central systolic blood pressure (cSBP) among hypertensive adults [32]. Cong et al. found that TyG index was associated with prehypertension and hypertension in the normoglycemic subjects (n = 32,124) [15].

Various studies have proved that TyG index trajectories are closely correlated with cardiovascular events. A study of 2480 individuals, enrolled from Hanzhong Adolescent Hypertension Cohort study, found that the highest TyG index trajectory carried the greatest odds of increased arterial stiffness, with a fully adjusted odds ratio (OR) of 2.76 (95% CI 1.40, 7.54) [33]. Another study demonstrated that participants with TyG index trajectories at high and very high levels had an even greater risk of future incident peripheral arterial disease (PAD) in the large-scale middle-aged adults [16]. Using the latent class trajectory modeling method, 10,380 adults with multiple general health checks were categorized to 3 distinct trajectories of the TyG index. Gao et al. further confirmed the moderate-stable trajectory of the TyG index was associated with carotid atherosclerosis (CAS) progression [34]. Moreover, one large prospective study had investigated the association of longitudinal pattern of the TyG index and stroke risk among patients with hypertension, and the results showed that high TyG index trajectory had a higher risk of stroke and ischemic stroke during the follow-up of 10 years [23]. Liao et al. and Wang et al. described the associations between TyG index trajectories and cardiovascular disease (CVD) in young adulthood and normal-weight adults [27, 35]. From all these present studies, we known the various TyG index trajectories could distinguish participants with similar patterns over a long period, it is a more effective approach to assess the association between the TyG index and potential effects on cardiovascular diseases. While a small number of studies have investigated the TyG index trajectory and hypertension risk in large prospective cohort. In the present study, we identified 3 distinct trajectories of TyG index by using GBTM, and we found that participants in “moderate increasing” and “high stable” trajectories were associated with higher hypertension risk. Thus, the findings of our study further confirmed TyG index is an important predictor of hypertension and relative diseases in the general population.

We also observed the strength of the association of TyG index trajectory and hypertension risk in the sex-stratified analysis. Our study observed that females had significant higher risk of hypertension in the “moderate increasing” and “high stable” TyG index trajectories than males. Another study based on middle-aged and older Chinese, using China Health and Retirement Longitudinal Study (CHARLS) data, also found that higher TyG index was significantly associated with stage 2 hypertension in females, but not in males [31]. However, previous study from Spanish cohort has reported that the top quintile of TyG index in men had two times more likely to develop hypertension than those in the bottom quintile, and the effect was attenuated in women [36]. For the inconsistent result of the association between TyG index and hypertension in sex differences need further investigation.

This study has several advantages. Our research is a longitudinal study based on an abundant sample of the physical examination population, they underwent regular health examination for several years, which provides a unique opportunity to examine the longitudinal TyG index trajectory patterns during follow-up over 12 years. In addition, the GBTM approach analysis distinguishes the long-term TyG index trajectory patterns, which could provide reliable and qualitative groups that individuals exhibited specific and similar TyG index changes over time. This grouping enabled robust classification and had more statistical power.

However, this study also has several limitations. First, our study enrolled individuals from the general population in northern China. Therefore, the identified trajectories could limit generalizability. Secondly, although several confounding factors we know that may have affected hypertension were enrolled in the model for adjustment, there are still unmeasured confounding components that may have influenced the results. Third, as we are retrospective cohort study, there exists an inevitable recall bias and missing data despite the fact that we had carefully adjusted the well-known and suspected risk factors.

## Conclusion

Our findings suggest that not only the baseline TyG index but also the high trajectories of TyG index growth were associated with the development of hypertension. Long-term monitoring of dynamic changes of TyG index may identify individuals at high risk of hypertension. Prevention programs that target TyG index may provide more effective for primary and secondary prevention of hypertension.

### Supplementary Information

**Additional file 1:** **Table S1.** Average probabilities of group assignment and Bay Information Criterion (BIC) statistics of model fits. **Table S2.** Baseline characteristics of participants according to quartiles of TyG index. **Table S3.** Baseline characteristics of participants included in the study compared to those who excluded for hypertension at baseline. **Figure S1.** Summary of missing data. **Figure S2.** Changes in standard mean differences (SMD) between different variables before and after propensity score matching (PSM). **Figure S3.** Incident rates of hypertension in every year. **Figure S4.** Comparison between groups for different sensitivity analysis. **Figure S5.** The restricted cubic spline curves for TyG index with risk of incident hypertension.

## Data Availability

The datasets used and analyzed in the current study are available from the corresponding author upon reasonable request.

## Abbreviations

TyG: Triglyceride glucose index
FBG: Fasting blood glucose
TC: Total cholesterol
HDL: High-densitylipoprotein
LDL: Low-densitylipoprotein
WBC: White blood cell
RBC: Red blood cell
NE: Neutrophils
PLT: Platelet
Hb: Haemoglobin
Cr: Creatinine
UA: Uric acid
HBP: High blood pressure
eGFR: Glomerular filtration rate
GBTM: Group-based trajectory modeling

## References

[1] Mills KT, Stefanescu A, He J (2020). The global epidemiology of hypertension. Nat Rev Nephrol.

[2] Ma S, Yang L, Zhao M, Magnussen CG, Xi B (2021). Trends in hypertension prevalence, awareness, treatment and control rates among Chinese adults, 1991–2015. J Hypertens.

[3] Mills KT, Bundy JD, Kelly TN, Reed JE, Kearney PM, Reynolds K, Chen J, He J (2016). Global disparities of hypertension prevalence and control: a systematic analysis of population-based studies from 90 countries. Circulation.

[4] Lewington S, Lacey B, Clarke R, Guo Y, Kong XL, Yang L, Chen Y, Bian Z, Chen J, Meng J (2016). The burden of hypertension and associated risk for cardiovascular mortality in China. JAMA Intern Med.

[5] Hypertension. 2023; https://www.who.int/news-room/fact-sheets/detail/hypertension. Accessed 1 July 2023.

[6] Hypertension in China. 2023. https://www.who.int/china/health-topics/hypertension.Accessed 1 July 2023.

[7] Zhou B, Carrillo-Larco RM, Danaei G, Riley LM, Paciorek CJ, Stevens GA, Gregg EW, Bennett JE, Solomon B, Singleton RK, Sophiea MK (2021). Worldwide trends in hypertension prevalence and progress in treatment and control from 1990 to 2019: a pooled analysis of 1201 population-representative studies with 104 million participants. Lancet..

[8] Roth GA, Abate D, Abate KH, Abay SM, Abbafati C, Abbasi N, Abbastabar H, Abd-Allah F, Abdela J, Abdelalim A, Abdollahpour I (2018). Global, regional, and national age-sex-specific mortality for 282 causes of death in 195 countries and territories, 1980–2017: a systematic analysis for the Global Burden of Disease Study 2017. Lancet..

[9] Gartlehner G, Vander Schaaf EB, Orr C, Kennedy SM, Clark R, Viswanathan M (2020). Screening for hypertension in children and adolescents: updated evidence report and systematic review for the US preventive services task force. JAMA.

[10] Wang Y, Yang W, Jiang X (2021). Association between triglyceride-glucose index and hypertension: a meta-analysis. Front Cardiovasc Med.

[11] Gao Q, Lin Y, Xu R, Luo F, Chen R, Li P, Zhang Y (2023). Positive association of triglyceride-glucose index with new-onset hypertension among adults: a national cohort study in China. Cardiovasc Diabetol.

[12] Zheng R, Mao Y (2017). Triglyceride and glucose (TyG) index as a predictor of incident hypertension: a 9-year longitudinal population-based study. Lipids Health Dis.

[13] Zhu B, Wang J, Chen K, Yan W, Wang A, Wang W, Gao Z, Tang X, Yan L, Wan Q (2020). A high triglyceride glucose index is more closely associated with hypertension than lipid or glycemic parameters in elderly individuals: a cross-sectional survey from the Reaction Study. Cardiovasc Diabetol.

[14] Liu XZ, Fan J, Pan SJ (2019). METS-IR, a novel simple insulin resistance indexes, is associated with hypertension in normal-weight Chinese adults. J Clin Hypertens (Greenwich).

[15] Zhang F, Zhang Y, Guo Z, Yang H, Ren M, Xing X, Cong H (2021). The association of triglyceride and glucose index, and triglyceride to high-density lipoprotein cholesterol ratio with prehypertension and hypertension in normoglycemic subjects: a large cross-sectional population study. J Clin Hypertens (Greenwich).

[16] Gao JW, Hao QY, Gao M, Zhang K, Li XZ, Wang JF, Vuitton DA, Zhang SL, Liu PM (2021). Triglyceride-glucose index in the development of peripheral artery disease: findings from the Atherosclerosis risk in communities (ARIC) study. Cardiovasc Diabetol.

[17] Barzegar N, Tohidi M, Hasheminia M, Azizi F, Hadaegh F (2020). The impact of triglyceride-glucose index on incident cardiovascular events during 16 years of follow-up: tehran lipid and glucose study. Cardiovasc Diabetol.

[18] Wu S, Xu L, Wu M, Chen S, Wang Y, Tian Y (2021). Association between triglyceride-glucose index and risk of arterial stiffness: a cohort study. Cardiovasc Diabetol.

[19] Wang A, Wang G, Liu Q, Zuo Y, Chen S, Tao B, Tian X, Wang P, Meng X, Wu S (2021). Triglyceride-glucose index and the risk of stroke and its subtypes in the general population: an 11-year follow-up. Cardiovasc Diabetol.

[20] Tian X, Zuo Y, Chen S, Liu Q, Tao B, Wu S, Wang A (2021). Triglyceride-glucose index is associated with the risk of myocardial infarction: an 11-year prospective study in the Kailuan cohort. Cardiovasc Diabetol.

[21] Miao M, Zhou G, Bao A, Sun Y, Du H, Song L, Cao Y, You S, Zhong C (2022). Triglyceride-glucose index and common carotid artery intima-media thickness in patients with ischemic stroke. Cardiovasc Diabetol.

[22] Wang X, Xu W, Song Q, Zhao Z, Meng X, Xia C, Xie Y, Yang C, Jin P, Wang F (2022). Association between the triglyceride-glucose index and severity of coronary artery disease. Cardiovasc Diabetol.

[23] Huang Z, Ding X, Yue Q, Wang X, Chen Z, Cai Z, Li W, Cai Z, Chen G, Lan Y (2022). Triglyceride-glucose index trajectory and stroke incidence in patients with hypertension: a prospective cohort study. Cardiovasc Diabetol.

[24] Zhang Y, Wang B (2023). Research progress on the applicability of estimating formula of glomerular filtration rate. Clin Res Pract.

[25] American Diabetes Association (2020). Standards of medical care in diabetes-2020 abridged for primary care providers. Clin Diabetes..

[26] Al-Makki A, DiPette D, Whelton PK, Murad MH, Mustafa RA, Acharya S, Beheiry HM, Champagne B, Connell K, Cooney MT (2022). Hypertension pharmacological treatment in adults: a world health organization guideline executive summary. Hypertension.

[27] Xu X, Huang R, Lin Y, Guo Y, Xiong Z, Zhong X, Ye X, Li M, Zhuang X, Liao X (2022). High triglyceride-glucose index in young adulthood is associated with incident cardiovascular disease and mortality in later life: insight from the CARDIA study. Cardiovasc Diabetol.

[28] Nagin DS, Odgers CL (2010). Group-based trajectory modeling in clinical research. Annu Rev Clin Psychol.

[29] Pan Y, Zou S, Xu Y, Di R, Gu H, Wang Z, Wei X, Yang C, Zhang G (2023). Is there any association between early trimester triglyceride-glucose index and incidence of hypertensive disorder of pregnancy and adverse pregnancy outcomes?. Front Endocrinol (Lausanne).

[30] Lee DH, Park JE, Kim SY, Jeon HJ, Park JH (2022). Association between the triglyceride-glucose (TyG) index and increased blood pressure in normotensive subjects: a population-based study. Diabetol Metab Syndr.

[31] Shan S, Li S, Lu K, Cao J, Sun W, Zhou J, Ren Z, Zhu S, Hou L, Chen D (2023). Associations of the Triglyceride and Glucose Index With hypertension stages, phenotypes, and their progressions among middle-aged and older chinese. Int J Public Health.

[32] Wang L, Cao TY, Li JQ, Ding CC, Li JP, Ying HB, Liu LS, Huang X (2022). Positive association between triglyceride glucose index and central systolic blood pressure among hypertensive adults. J Geriatr Cardiol..

[33] Yan Y, Wang D, Sun Y, Ma Q, Wang K, Liao Y, Chen C, Jia H, Chu C, Zheng W (2022). Triglyceride-glucose index trajectory and arterial stiffness: results from hanzhong adolescent hypertension cohort study. Cardiovasc Diabetol.

[34] Yu H, Tao L, Li YG, Yang L, Liu D, Wang Y, Hao X, He H, Che Y, Wang P (2023). Association between triglyceride-glucose index trajectories and carotid atherosclerosis progression. Cardiovasc Diabetol.

[35] Tian X, Zuo Y, Chen S, Meng X, Chen P, Wang Y, Wu S, Luo Y, Wang A (2022). Distinct triglyceride-glucose trajectories are associated with different risks of incident cardiovascular disease in normal-weight adults. Am Heart J..

[36] Sánchez-Íñigo L, Navarro-González D, Pastrana-Delgado J, Fernández-Montero A, Martínez JA (2016). Association of triglycerides and new lipid markers with the incidence of hypertension in a Spanish cohort. J Hypertens.

