# Supplementary material for: The triglyceride glucose index trajectory is associated with hypertension: a retrospective longitudinal cohort study
Source: Cardiovasc Diabetol. 2023 Dec 15;22:347. doi: 10.1186/s12933-023-02087-w (PMC10725029; doi:10.1186/s12933-023-02087-w)
Supplement: Supplementary file 1 — Additional file 1: Table S1. Average probabilities of group assignment and Bay Information Criterion (BIC) statistics of model fits. Table S2. Baseline characteristics of participants according to quartiles of TyG index. Table S3. Baseline characteristics of participants included in the study compared to those who excluded for hypertension at baseline. Figure S1. Summary of missing data. Figure S2. Changes in standard mean differences (SMD) between different variables before and after propensity score matching (PSM). Figure S3. Incident rates of hypertension in every year. Figure S4. Comparison between groups for different sensitivity analysis. Figure S5. The restricted cubic spline curves for TyG index with risk of incident hypertension. [file 12933_2023_2087_MOESM1_ESM.docx]

**SUPPLEMENTARY MATERIALS**

**Supplementary Table 1. Average probabilities of group assignment and Bay Information Criterion (BIC) statistics of model fits**

|  | **Group1** | **Group2** | **Group3** | **Group4** | **Group5** | **BIC for total number of observations (N = 59126)** | **BIC for total participants (N = 15056)** | **AIC** | **Entropy** | **% Participants per** **trajectory group** |
| --- | --- | --- | --- | --- | --- | --- | --- | --- | --- | --- |
|  |  |  |  |  |  |  |  |  |  |  |
| 2 groups | 0.96 | 0.92 |  |  |  | -40313.79 | -40306.95 | -40268.85 | 0.82 | 69.42/30.58 |
| **3 groups** | **0.93** | **0.90** | **0.93** |  |  | **-36027.03** | **-36016.77** | **-35959.62** | **0.82** | **47.76/42.92/9.32** |
| 4 groups | 0.89 | 0.86 | 0.88 | 0.93 |  | -34238.67 | -34226.36 | -34157.78 | 0.79 | 31.49/42.82/21.43/4.26 |
| 5 groups | 0.86 | 0.83 | 0.85 | 0.89 | 0.05 | 33406.23 | -33389.81 | -33298.38 | 0.78 | 24.36/40.32/26.11/8.02/1.19 |

**Supplementary Table 2. Baseline characteristics of participants according to quartiles of TyG index**

| **Variables** | **Total** | **Q1** | **Q2** | **Q3** | **Q4** | ***p*** |
| --- | --- | --- | --- | --- | --- | --- |
| n | 15,056 | 3,764 | 3,764 | 3,764 | 3,764 |  |
| Age, years^a^ | 38.00 (30.00-47.00) | 33.00 (28.00-41.00) | 37.00 (30.00-46.00) | 40.00 (31.00-49.00) | 44.00 (34.00-51.00) | < 0.01 |
| Male (%)^b^ | 7352 (48.83) | 947 (25.16) | 1488 (39.53) | 2136 (56.75) | 2781 (73.88) | < 0.01 |
| TyG index^a^ | 8.44 (8.08-8.85) | 7.86 (7.70-7.98) | 8.26 (8.17-8.35) | 8.63 (8.52-8.73) | 9.15 (8.98-9.42) | 0.00 |
| SBP (mmHg)^a^ | 120.00 (111.00-128.00) | 115.00 (107.00-124.00) | 118.00 (109.00-126.00) | 121.00 (112.00-129.00) | 124.00 (116.00-131.00) | < 0.01 |
| DBP (mmHg)^a^ | 72.00 (66.00-79.00) | 69.00 (63.00-75.00) | 71.00 (65.00-77.00) | 73.00 (67.00-80.00) | 76.00 (70.00-82.00) | < 0.01 |
| Height (cm)^a^ | 168.00 (162.00-174.00) | 165.00 (161.00-171.00) | 167.00 (162.00-173.00) | 169.00 (163.00-175.00) | 172.00 (166.00-177.00) | < 0.01 |
| Weight (kg)^a^ | 66.00 (58.00-76.00) | 58.00 (53.00-65.00) | 63.00 (56.00-71.00) | 69.00 (61.00-77.00) | 76.00 (67.13-84.00) | 0.00 |
| BMI (kg/cm²)^a^ | 23.41 (21.22-25.82) | 21.48 (19.81-23.25) | 22.63 (20.70-24.68) | 24.07 (22.13-26.15) | 25.66 (23.72-27.75) | 0.00 |
| WBC (10^9/L)^a^ | 6.01 (5.12-7.05) | 5.57 (4.77-6.51) | 5.81 (4.98-6.80) | 6.15 (5.24-7.17) | 6.52 (5.61-7.63) | < 0.01 |
| RBC (10^12/L)^a^ | 4.75 (4.45-5.10) | 4.51 (4.29-4.80) | 4.66 (4.41-5.00) | 4.85 (4.52-5.16) | 5.02 (4.72-5.28) | 0.00 |
| NE (10^9/L)^a^ | 3.37 (2.73-4.13) | 3.11 (2.51-3.82) | 3.27 (2.64-3.99) | 3.46 (2.84-4.21) | 3.64 (3.03-4.47) | < 0.01 |
| Hb (g/L)^a^ | 143.00 (132.00-155.00) | 135.00 (127.00-143.00) | 139.00 (131.00-151.00) | 147.00 (135.00-157.00) | 153.00 (142.00-161.00) | 0.00 |
| Alb (g/L)^a^ | 46.40 (44.70-48.10) | 46.10 (44.42-47.80) | 46.30 (44.60-47.96) | 46.50 (44.80-48.10) | 46.90 (45.14-48.58) | < 0.01 |
| PLT (10^9/L)^a^ | 224.00 (194.00-259.00) | 221.00 (192.00-253.00) | 226.00 (194.00-261.00) | 225.00 (195.00-261.00) | 225.00 (194.00-260.00) | < 0.01 |
| UREA (mmol/L)^a^ | 4.66 (3.91-5.47) | 4.46 (3.73-5.29) | 4.53 (3.82-5.38) | 4.74 (3.96-5.50) | 4.85 (4.12-5.65) | < 0.01 |
| Cr (µmol/L)^a^ | 65.00 (54.56-76.00) | 57.86 (51.00-67.99) | 62.00 (53.72-74.00) | 67.80 (56.01-78.00) | 71.00 (61.00-79.65) | < 0.01 |
| UA (µmol/L)^a^ | 315.03 (257.00-385.00) | 265.00 (228.00-316.00) | 295.00 (248.00-356.00) | 333.00 (274.90-393.00) | 378.44 (318.69-439.34) | 0.00 |
| FBG (mmol/L)^a^ | 5.36 (5.08-5.69) | 5.12 (4.90-5.37) | 5.29 (5.04-5.56) | 5.43 (5.17-5.73) | 5.65 (5.34-6.09) | 0.00 |
| CHOL (mmol/L)^a^ | 4.69 (4.16-5.27) | 4.31 (3.87-4.79) | 4.59 (4.12-5.10) | 4.81 (4.28-5.37) | 5.10 (4.57-5.71) | 0.00 |
| TG (mmol/L)^a^ | 1.06 (0.77-1.57) | 0.63 (0.54-0.70) | 0.91 (0.83-1.00) | 1.28 (1.16-1.42) | 2.07 (1.76-2.63) | 0.00 |
| HDL (mmol/L)^a^ | 1.29 (1.09-1.52) | 1.48 (1.30-1.69) | 1.35 (1.18-1.57) | 1.23 (1.07-1.42) | 1.08 (0.95-1.26) | 0.00 |
| LDL (mmol/L)^a^ | 2.66 (2.21-3.16) | 2.33 (1.97-2.77) | 2.62 (2.22-3.08) | 2.84 (2.39-3.32) | 2.89 (2.43-3.41) | < 0.01 |
| eGFR (ml/min/m³)^a^ | 111.67 (100.36-123.67) | 116.94 (106.07-129.63) | 112.52 (101.52-123.72) | 109.46 (98.22-121.37) | 108.01 (96.84-119.81) | < 0.01 |
| diabetes, n (%)^b^ | 252 (1.67) | 2 (0.05) | 16 (0.42) | 36 (0.96) | 198 (5.26) | < 0.01 |
| Smoking (%)^b^ | 597 (3.96) | 58 (1.54) | 96 (2.55) | 173 (4.60) | 270 (7.17) | < 0.01 |
| Drinking (%)^b^ | 63 (0.42) | 4 (0.11) | 10 (0.26) | 17 (0.45) | 32 (0.85) | < 0.01 |
| family history  of hypertension (%)^b^ | 698 (4.64） | 151 (4.01) | 160 (4.25) | 199 (5.29) | 188 (4.99) | 0.03 |
| family history  of diabetes (%)^b^ | 406 (2.70) | 64 (1.70) | 91 (2.42) | 102 (2.71) | 149 (3.96) | < 0.01 |

Q, quartile,BMI, body mass index; HDL, high density lipoprotein; LDL, low density lipoprotein; SBP, systolic blood pressure; DBP, diastolic blood pressure; WBC, white blood cell; RBC, red blood cell; NE, neutrophils; Cr, creatinine; Hb, haemoglobin; Alb, albumin; PLT, platelet; CHOL, cholesterol; TG, triglyceride glucose; FPG, fasting plasma glucose; TyG, Triglyceride glucose index = In (fasting TG (mg/dL) × fasting plasma glucose [mg/Dl]/ 2 ), eGFR Glomerular filtration rate.

^a^ Data are given as median (interquartile range).

^b^ Data are expressed as number (percentage).

**Table S3 Baseline characteristics of participants included in the study compared to those who excluded for hypertension at baseline**

| **Variables** | **Overall** | **Included** | **Excluded for hypertension** | ***p*** |
| --- | --- | --- | --- | --- |
| n | 20,874 | 15,056 | 5,818 |  |
| Gender (man/women)^b^ | 11,750/9,124 | 7,352/7,704 | 4,398/1,420 | < 0.01 |
| Age (year)^a^ | 42.00 (32.00-51.00) | 38.00 (30.00-47.00) | 51.00 (42.00-59.00) | < 0.01 |
| TyG index^a^ | 8.56 (8.17-9.00) | 8.44 (8.08-8.85) | 8.90 (8.54-9.31) | 0.00 |
| SBP (mmHg)^a^ | 125.00 (114.00-138.00) | 120.00 (111.00-128.00) | 147.00 (140.00-158.00) | 0.00 |
| DBP (mmHg)^a^ | 76.00 (68.00-85.00) | 72.00 (66.00-79.00) | 90.00 (83.00-97.00) | 0.00 |
| Height (cm)^a^ | 169.00 (163.00-175.00) | 168.00 (162.00-174.00) | 171.00 (164.38-176.00) | < 0.01 |
| Weight (kg)^a^ | 69.00 (60.00-79.00) | 66.00 (58.00-76.00) | 76.00 (67.90-85.00) | 0.00 |
| BMI (kg/cm²)^a^ | 24.22 (21.76-26.67) | 23.41 (21.22-25.82) | 26.12 (23.95-28.37) | 0.00 |
| WBC (10^9/L)^a^ | 6.12 (5.21-7.19) | 6.01 (5.12-7.05) | 6.42 (5.48-7.49) | < 0.01 |
| RBC (10^12/L)^a^ | 4.83 (4.50-5.16) | 4.75 (4.45-5.10) | 4.99 (4.69-5.26) | < 0.01 |
| NE (10^9/L)^a^ | 3.43 (2.79-4.22) | 3.37 (2.73-4.13) | 3.63 (2.95-4.42) | < 0.01 |
| Hb (g/L)^a^ | 146.00 (134.00-157.00) | 143.00 (132.00-155.00) | 153.00 (143.00-161.00) | < 0.01 |
| PLT (10^9/L)^a^ | 222.00 (191.00-257.00) | 224.00 (194.00-259.00) | 215.00 (185.00-250.00) | < 0.01 |
| Alb (g/L)^a^ | 46.44 (44.71-48.10) | 46.40 (44.70-48.10) | 46.50 (44.78-48.20) | 0.24 |
| TP (g/L)^a^ | 72.10 (69.5-75) | 72.00 (69.30-75.00) | 72.40 (69.80-75.30) | < 0.01 |
| UREA (mmol/L)^a^ | 4.79 (4.03-5.62) | 4.66 (3.91-5.47) | 5.13 (4.40-5.99) | < 0.01 |
| Cr (µmol/L)^a^ | 67.00 (56.00-77.00) | 65.00 (54.56-76.00) | 71.00 (62.00-79.99) | < 0.01 |
| UA (µmol/L)^a^ | 331.00 (267.66-399.00) | 315.03 (257.00-385.00) | 366.00 (307.47-426.94) | < 0.01 |
| FBG (mmol/L)^a^ | 5.46 (5.14-5.86) | 5.36 (5.08-5.69) | 5.82 (5.42-6.47) | 0.00 |
| CHOL (mmol/L)^a^ | 4.78 (4.23-5.40) | 4.69 (4.16-5.27) | 5.05 (4.45-5.69) | < 0.01 |
| TG (mmol/L)^a^ | 1.18 (0.82-1.74） | 1.06 (0.77-1.57) | 1.53 (1.08-2.18) | 0.00 |
| HDL (mmol/L)^a^ | 1.25 (1.06-1.48） | 1.29 (1.09-1.52) | 1.17 (1.01-1.37) | < 0.01 |
| LDL (mmol/L)^a^ | 2.72 (2.25-3.24） | 2.66 (2.21-3.16) | 2.91 (2.41-3.46) | < 0.01 |

BMI, body mass index; HDL, high density lipoprotein; LDL, low density lipoprotein; SBP, systolic blood pressure; DBP, diastolic blood pressure; WBC, white blood cells; RBC, red blood cell; NE, neutrophils; Hb, haemoglobin; Alb, albumin; PLT, platelet; CHOL, cholesterol; Cr, creatinine; UA, uric acid; TC, total cholesterol; FPG, fasting plasma glucose; TG, triglyceride glucose; TyG, Triglyceride glucose index = ln[fasting TG (mg/dL) × fasting plasma glucose (mg/dL)/ 2 ].

^a^ Data are given as median (interquartile range).

^b^ Data are expressed as number (percentage)

**Supplementary Figure 1. Summary of missing data**


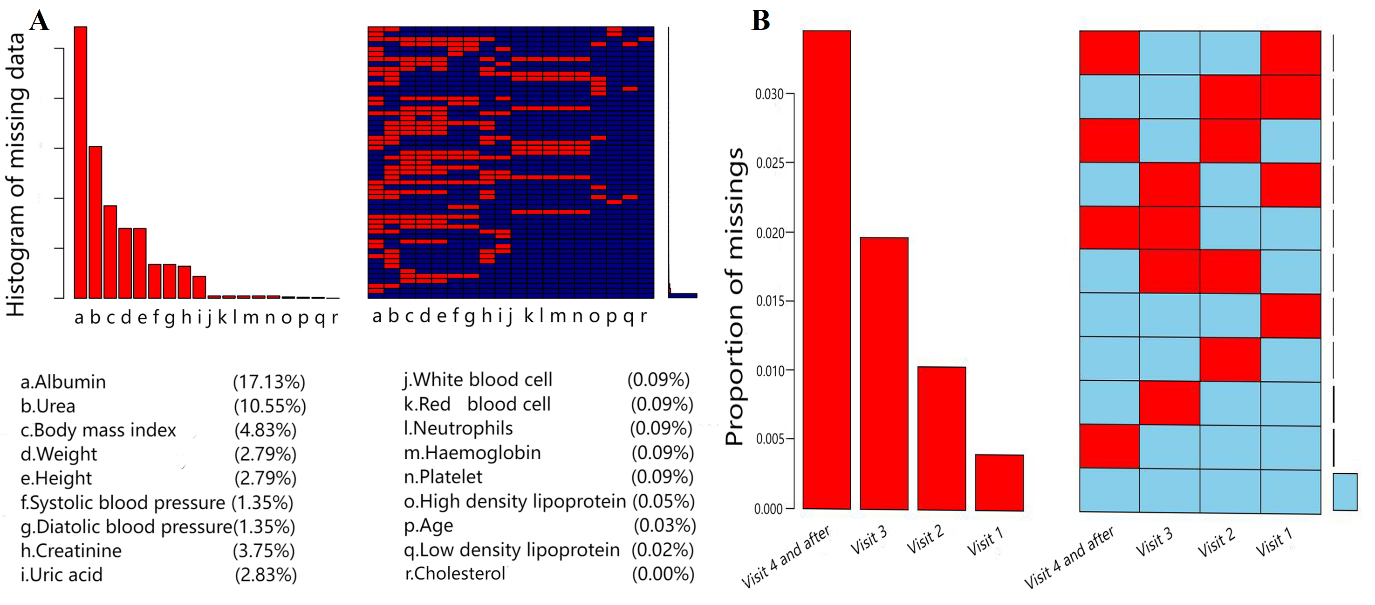


A. represents all missing variables; B. represent the absence of blood pressure values during follow-up periods.

**Supplementary Figure 2. Changes in standard mean differences (SMD) between different variables before and after propensity score matching (PSM)**

**
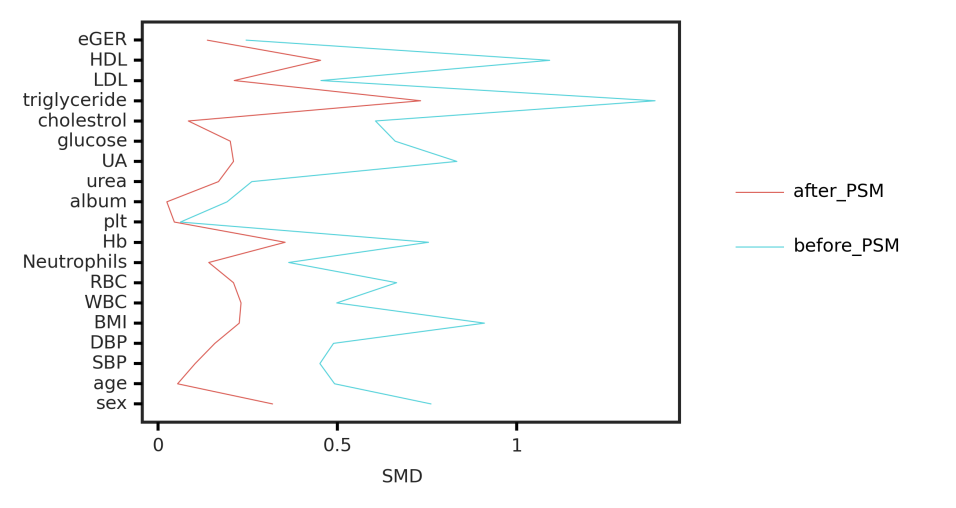
**

**Supplementary Figure 3. Incident rates of hypertension in every year**

**
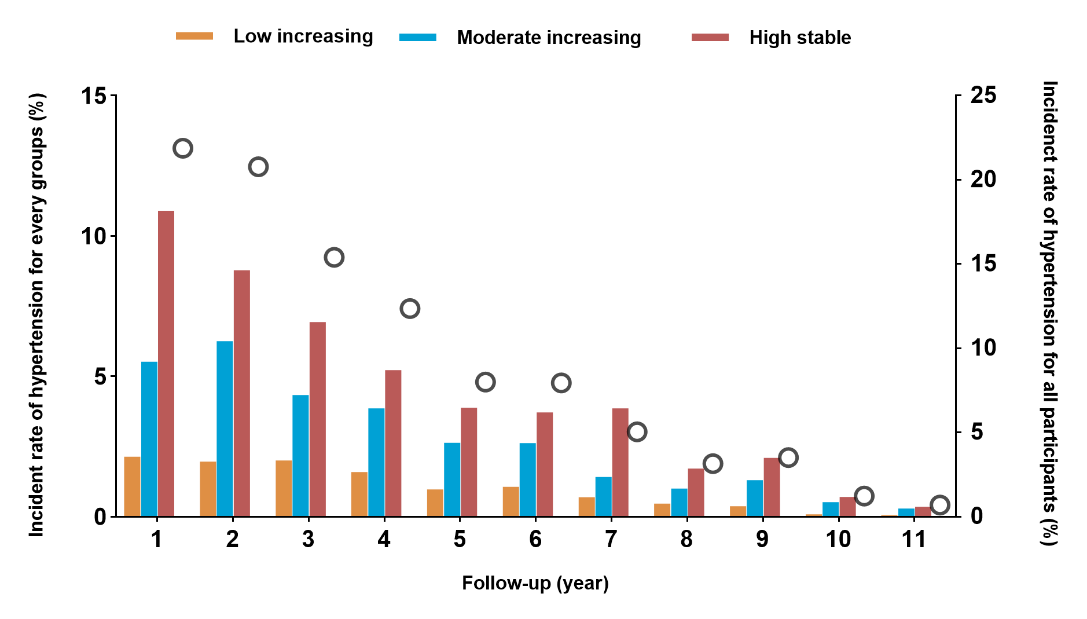
**

Histogram stand for incident rate of hypertension for three trajectory groups in every year (left ordinate). Circles stand for incident rate of hypertension for all participants in every follow-up year (right ordinate)

**Supplementary Figure 4. Comparison between groups for different sensitivity analysis**

**
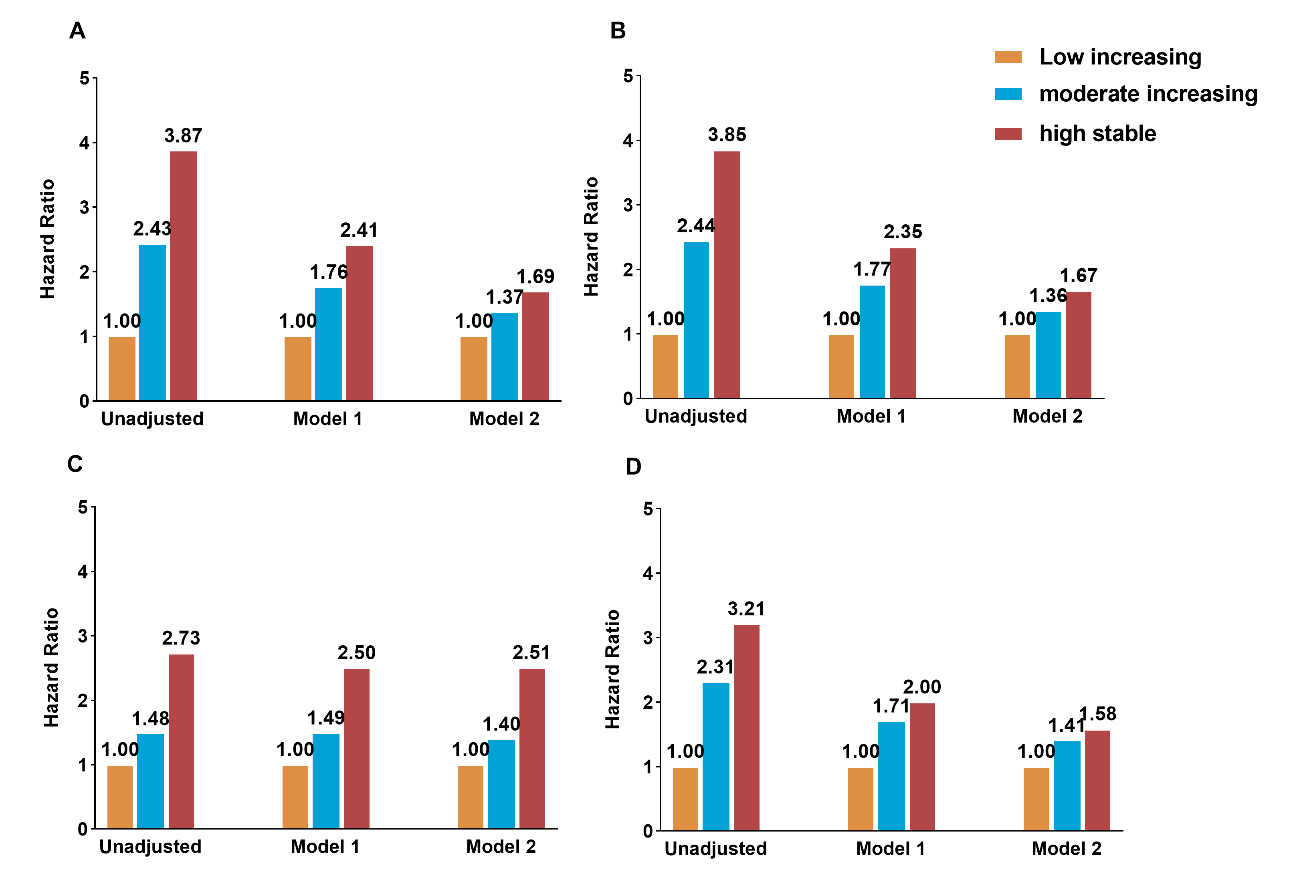
**

A: participants who were on any antidiabetic, or lipid lowering medications were excluded; B: completing the missing data with multiple imputation; C: data disposed by multi-group propensity scores; D: participants who developed hypertension during the second follow-up were excluded.

Model 1: adjusted for baseline age and sex.

Model 2 :further adjusted for model 1 covariates plus baseline diabetes, smoking, systolic blood pressure, diastolic blood pressure, BMI, high density lipoprotein cholesterol, low density lipoprotein cholesterol, blood glucose, triglyceride, cholesterol, urea, uric acid, glomerular filtration rate.

**Supplementary Figure 5. The restricted cubic spline curves for TyG index with risk of incident hypertension**

**
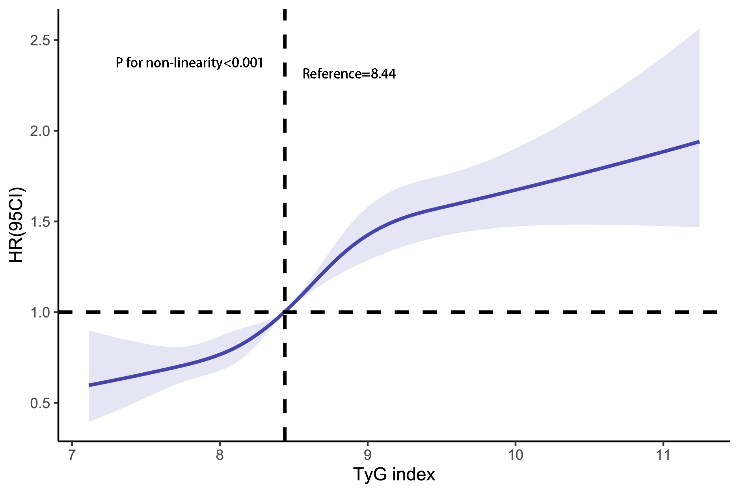
**

Data were fitted with a linear regression model using restricted cubic splines with 5 knots at 5, 25, 50,75, and 95th percentiles of TyG index. The model was adjusted for age, sex. The solid dark blue line represented the estimations, and the shaded part represented 95% confidence interval
